# Identification of an Unconventional Subpeptidome Bound to the Behçet's Disease-associated HLA-B*51:01 that is Regulated by Endoplasmic Reticulum Aminopeptidase 1 (ERAP1)*[Fn FN2]

**DOI:** 10.1074/mcp.RA119.001617

**Published:** 2020-03-11

**Authors:** Liye Chen, Hui Shi, Danai Koftori, Takuya Sekine, Annalisa Nicastri, Nicola Ternette, Paul Bowness

**Affiliations:** ‡ Nuffield Department of Orthopaedics, Rheumatology and Musculoskeletal Sciences, University of Oxford, Oxford, UK; § Jenner Institute, University of Oxford, Oxford, UK; ¶ Target Discovery Institute, University of Oxford, Oxford, UK

**Keywords:** Immunology, Tandem Mass Spectrometry, Immunoaffinity, Mass Spectrometry, Peptides, Antigen Presentation, Behçet's Disease, ERAP1, HLA-B*51:01, Immunopeptidome

## Abstract

Human leukocyte antigen (HLA) B*51:01 and endoplasmic reticulum aminopeptidase 1 (ERAP1) are strongly genetically associated with Behçet's disease (BD). Previous studies have defined two subgroups of HLA-B*51 peptidome containing proline (Pro) or alanine (Ala) at position 2 (P2). Little is known about the unconventional non-Pro/Ala2 HLA-B*51-bound peptides. We aimed to study the features of this novel subpeptidome, and investigate its regulation by ERAP1. CRISPR-Cas9 was used to generate an HLA-ABC-triple knockout HeLa cell line (HeLa.ABC-KO), which was subsequently transduced to express HLA-B*51:01 (HeLa.ABC-KO.B51). ERAP1 was silenced using lentiviral shRNA. Peptides bound to HLA-B*51:01 were eluted and analyzed by mass spectrometry. The characteristics of non-Pro/Ala2, Pro2, and Ala2 peptides and their alteration by ERAP1 silencing were investigated. Effects of ERAP1 silencing on cell surface expression of HLA-B*51:01 were studied using flow cytometry. More than 20% of peptides eluted from HLA-B*51:01 lacked Pro or Ala at P2. This unconventional group of HLA-B*51:01-bound peptides was relatively enriched for 8-mers (with relatively fewer 9-mers) compared with the Pro2 and Ala2 subpeptidomes and had similar N-terminal and C-terminal residue usages to Ala2 peptides (with the exception of the less abundant leucine at position Ω). Knockdown of ERAP1 increased the percentage of non-Pro/Ala2 from 20% to ∼40%, increased the percentage of longer (10-mer and 11-mer) peptides eluted from HLA-B*51:01 complexes, and abrogated the predominance of leucine at P1. Interestingly knockdown of ERAP1 altered the length and N-terminal residue usage of non-Ala2&Pro2 and Ala2 but not the Pro2 peptides. Finally, ERAP1 silencing regulated the expression levels of cell surface HLA-B*51 in a cell-type-dependent manner. In conclusion, we have used a novel methodology to identify an unconventional but surprisingly abundant non-Pro/Ala2 HLA-B*51:01 subpeptidome. It is increased by knockdown of ERAP1, a gene affecting the risk of developing BD. This has implications for theories of disease pathogenesis.

## Abstract (precis)

HLA-B*51 and ERAP1 affect the risk of developing Behçet's disease (BD). Regulation of Pro2 and Ala2 HLA-B*51-bound peptides by ERAP1 has been studied but not the non-Pro/Ala2 ones. Through an HLA-ABC-triple knockout cell model that only expresses HLA-B*51, we show that the non-Pro/Ala2 peptides constitute around 20% of HLA-B*51-bound peptides, more abundant than previously known. This subpeptidome has unique features in peptide length and motif. ERAP1 silencing increases the abundance of non-Pro/Ala2 subpeptidome and alters its length and peptide motif.

## Graphical Abstract

**Figure:**
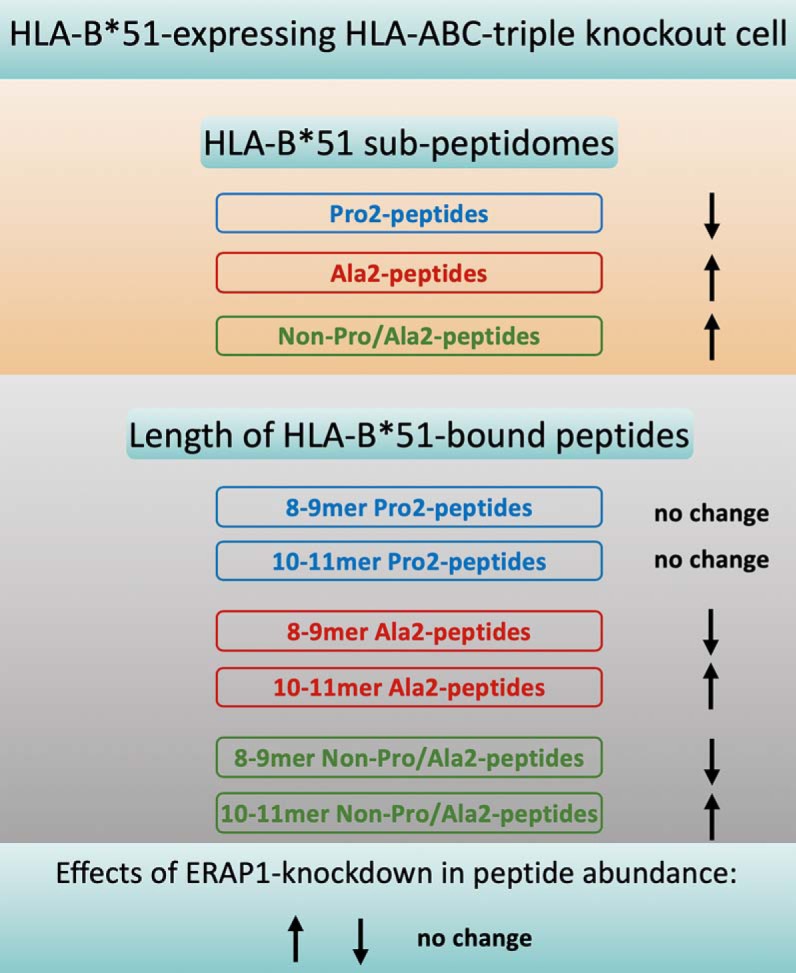

- A novel HLA-ABC-triple knockout cell model to study the HLA-B*51 peptidome.
- Enrichment of the unconventional non-Pro/Ala2 HLA-B*51 peptides following ERAP1 silencing.
- Knockdown of ERAP1 increases the length of non-Pro/Ala2 and Ala2 peptides, not that of Pro2.
- ERAP1 regulation of HLA-B*51 cell surface expression is cell type dependent.

BD^1^ is a chronic inflammatory disorder characterized by inflammatory arthritis, recurrent oral ulcers, ocular involvement, genital ulcers, and skin lesions (reviewed in (1)). While possession of HLA-B*51 is a strong genetic predisposing factor in BD, its pathogenic mechanism is still unclear. Recent genetic studies have identified ERAP1 as an additional gene associated with BD. Interestingly, ERAP1 polymorphisms only affect BD risk in HLA-B*51-positive individuals (2, 3). Similar epistatic interactions between HLA class I and ERAP1 have been reported in HLA-B*27-associated ankylosing spondylitis and HLA-C*0602-associated psoriasis (4, 5). The concept of “MHC-I-opathy” has been recently proposed for this group of diseases, including BD, sharing genetic association with MHC-I alleles and tissue-specific inflammation (6, 7).

ERAP1 locates in the ER and trims peptides to optimal length (usually 8–9 amino acids) prior to their binding to MHC class I molecules (8). ERAP1 cleaves amino acids on the N terminus of peptides but spares the peptides with Pro at position 2 (9). We have previously shown that silencing of ERAP1 increased the percentage of longer peptides (11–13 mers) bound to HLA-B*27 and reduced the presentation of an HLA-B*27-restricted HIV epitope (KK10) (10). Additionally, a loss-of-function ERAP1 variant, K528R, was reported to significantly alter the HLA-B*27 peptidome (11). We also observed reduced cell surface expression of HLA-B*27 β2-microglobulin (β2m)-FHCs following ERAP1 silencing or inhibition (12).

Pro and Ala are previously described position 2 (P2) anchor residues of the HLA-B*51:01-associated peptidome (13, 14), and studies of the regulation of HLA-B*51:01 bound peptides by ERAP1 have focused exclusively on these Pro2- and Ala2- peptides (14, 15). Little is known about the nature of non-Pro/Ala2 HLA-B*51:01-bound peptides or their ERAP1 dependence.

Using a novel HLA-A, B, and C knockout HeLa cell line expressing only HLA-B*51:01, we here describe a non-Pro/Ala2 subpeptidome comprising ∼20% of HLA-B*51:01-bound peptides. This unconventional subpeptidome differs from Pro2 and Ala2 peptides in peptide length and/or motif. We also show that ERAP1 knockdown, mimicking the loss-of-function ERAP1 variant predisposing to BD, increases the frequency of non-Pro/Ala2 peptides and reduces that of Pro2 peptides. Moreover, silencing of ERAP1 affects the length and motif of Ala2 and non-Pro/Ala2, but not Pro2 HLA-B*51:01-bound peptides. Finally, we show that ERAP1 regulates cell surface expression of HLA-B*51 in a cell-type-dependent manner.

## EXPERIMENTAL PROCEDURES

### 

#### 

##### Generation of Cell Lines

A previously described CRISPR-Cas9 plasmid, pSpCas9(BB)-2A-GFP (PX458), was acquired from Addgene (16). sgRNAs for HLA-A, HLA-B, and HLA-C were cloned into the plasmid (HLA-A: CACCGACAGCGACGCCGCGAGCCAG and AAACCTGGCTCGCGGCGTCGCTGTC, HLA-B: CACCGGGTTCTATCTCCTGCTGGTC and AAACGACCAGCAGGAGATAGAACCC, HLA-C: CACCGCGGACTGGTCATACCCGCGG and AAACCCGCGGGTATGACCAGTCCGC). The PX458-HLA-A, PX458-HLA-B, and PX458-HLA-C plasmids were sequentially transfected into HeLa cells using Genejuice (Merck, UK).

HLA-ABC-knockout HeLa cells were isolated through cell sorting of W6/32-negative cells. HLA-B*51:01 was then expressed using lentivirus to generate the HeLa.ABC-KO.B51 cells (pHR-SIN-B*51, p8.91, and pMDG plamids used for the production of lentivirus). Lymphoblastoid cell line 721.221, hereafter termed 221, and C1R cells expressing HLA-B*51:01 were generated in a previous study (17). All cell lines were cultured in RPMI 1640 supplemented with 10% fetal bovine serum, 0.1 mg/ml of streptomycin, and 100 units/ml of penicillin (R10).

##### Isolation of HLA-B*51-bound Peptides

1–5 × 10^8^ Cells were lysed in 5 ml of lysis buffer (0.5% Igepal, 150 mm NaCl, 50 mm Tris, pH 8.0, supplemented with cOmplete™ protease inhibitor mixture ((Merck)). HLA complexes were immunoprecipitated using 1-mg W6/32 antibody (pan-specific for HLA class I molecules) cross-linked to Protein G Sepharose beads (Fisher Scientific, UK) for purification of intact HLA-B*51 complexes. The beads were washed three times using 2 × 150 mm NaCl and 1 × 450 mm NaCl in 50 mm Tris buffer, and salt was removed in a final wash with 50 mm Tris. HLA peptides were eluted by addition of 2.5 ml 10% acetic acid. The enriched HLA-B*51 peptides were further purified from larger complex components by high-performance liquid chromatography (Ultimate 3000) on a ProSwift RP-1S 4,6 × 50 mm column (Thermo Scientific, UK) applying a linear gradient of 2–35% acetonitrile over 10 min. Alternating fractions that did not contain beta-2-microglobulin or alpha chain were pooled into three final fractions and further concentrated and kept at −80 °C prior to MS analysis.

##### Mass Spectrometry and Data Analysis

Peptides were suspended in 20 μl buffer A (1% acetonitrile, 0.1% TFA in water) and analyzed by nano-ultra-performance liquid chromatography coupled to high-resolution mass spectrometry using an Ultimate 3000 RSLCnano System coupled with an Orbitrap Fusion Lumos Tribrid mass spectrometer (Thermo Scientific). 9 μl of each sample were injected and trapped onto a 3-μm particle size 0.075 mm × 150 mm Acclaim PepMap RSLC column at 8 μl/min flowrate. Peptide separation was performed at 40 °C by applying a linear gradient of 3–25% buffer B (0.1% formic acid, 5% DMSO in acetonitrile) in buffer A (0.1% formic acid, 5% DMSO in water) over 1 h at a flow rate of 250 μl/min on a 2-μm particle size, 75 μm × 50 cm Acclaim PepMap RSLC column. Peptides were introduced to a Fusion Lumos mass spectrometer (Thermo Scientific) via an Easy-Spray source at 2000 V. The ion transfer tube temperature was set to 305 °C. Measurement of precursor peptides was performed with a resolution of 120,000 for full MS (300–1500 *m/z* scan range) at an automatic gain control target of 400,000. Precursor ion selection and fragmentation by high-energy collisional dissociation (at 28% collision energy for charge state 2–4, 35% for charge state 1) was performed in TopSpeed mode at an isolation window of 1.2 Da for singly to quarterly charged ions at a resolution of 30,000 and an AGC target of 300,000 in the Orbitrap for a cycle duration of 2 s. Singly charged ions were acquired with lower priority. MS data were analyzed with Peaks v7.5 *de novo* assisted database search (Bioinformatics Solutions, release date 16/06/2015) for identification of peptide sequences. Spectra were matched to all human reviewed 20210 SwissProt entries from March 3, 2016. The false discovery rate was estimated by simultaneous searching of a randomized database using the decoy-fusion approach (18), which is integrated in the Peaks software. The results were filtered using a score cutoff of -lg10P = 15, resulting in a false discovery rate of an average of 2.4%. The searches were performed with the following parameters: no enzyme specificity, no static and variable modifications, peptide tolerance: ± 5ppm and fragment tolerance: ± 0.03 Da.

##### ERAP1 Silencing Using shRNA/siRNA

HeLa.ABC-KO.B51 cells were stably transduced with a previously described lentiviral ERAP1-shRNA (10). 221.B51 and C1R.B51 cells were transiently transfected with ctr-siRNA or ERAP1-siRNA using Neon transfection kit (Thermo Fisher). A previously reported ERAP1 siRNA (AACGUAGUGAUGGGACACCAU-dTdT and AUGGUGUCCCAUCACUACG-dTdT) was used (8).

##### Flow Cytometry

W6/32 antibody (mouse IgG2a, specific for classical β2m-associated HLA-A, B, C, and E) and HC-10 antibody (mouse IgG2a, specific for HLA class I FHCs) were used to stain surface-expressed classical HLA-B*51 complexes and β2m FHCs in HeLa.ABC-KO.B51(19). APC-conjugated anti-mouse IgG antibody was used as secondary antibody. Dead cells were excluded using LIVE/DEAD® Fixable Violet Dead Cell Stain Kit (Thermo Fisher). BD LSRFortessa™ and Diva software were used. The latter converts channel value into fluorescence intensity using a logarithmic algorithm; therefore, geometric mean fluorescence intensity was used to quantify staining intensity.

##### Experimental Design and Statistical Rationale

The current study aims to: (1) determine the characteristics of unconventional non-Pro/Ala2 HLA-B*51 subpeptidome; (2) investigate the regulation of non-Pro/Ala2 HLA-B*51 subpeptidome by ERAP1; and (3) study the contribution of ERAP1 to HLA-B*51 cell surface expression.

For the first aim, two independent experiments were performed using the HLA-A, B, and C triple-knockout HeLa cell line that stably expresses HLA-B*51 (HeLa.ABC-KO.B51). For the second aim, HLA-B*51-bound peptides were isolated and sequenced using ERAP1-competent and ERAP1-deficient HeLa.ABC-KO.B51 cell lines. Two independent experiments were performed. To minimize the experimental artifacts, for each experiment, peptides from ERAP1-competent and ERAP1-dificient cell lines were prepared and sequenced on the same day. For the third aim, three HLA-B*51-expressing cell lines, HeLa.ABC-KO.B51, 221.B51, and C1R.B51, were transfected with either control siRNA or ERAP1 siRNA. Experiments were repeated three times for each cell line.

Chi-squared test with Bonferroni correction was used to compare the length distribution, P1 and C-terminal residue frequencies between Pro2, Ala2, and non-Pro/Ala2 peptides (Fig. 2 and Fig. S3), the length distribution, P2 and P1 residue frequencies of HLA-B*51-bound peptides between ERAP1-competent and ERAP1-silenced cells (Fig. 3*B* and Fig. 4), the length distribution, P1 residue frequency of Pro2, Ala2, and non-Pro/Ala2 peptides between ERAP1-competent and ERAP1-silenced cells (Fig. 5, Fig. 6, and Fig. S5). In Fig. 7, the paired two-tailed *t* test was used to compare expressional levels of HLA-B*51. A *p* value <0.05 was considered significant.

In this study, we have two biological replicates for peptidome experiments. The chi-squared test with Bonferroni correction has been used in previous peptidome studies (14, 15) and was selected here for the statistical comparison of peptide length and amino acid usage between different samples.

## RESULTS

### 

#### 

##### Generation of HeLa.ABC-KO.B51 and ERAP1-knockdown Cell Lines

In order to precisely study peptides bound to HLA-B*51:01, either a specific antibody or a cell line expressing only HLA-B*51:01 is required. W6/32, the most commonly used HLA class I antibody, binds HLA-B*51:01 but also reacts with most other HLA class I molecules, which are expressed even in HLA class I-low cell lines such as 721.221 and C1R cells (supplemental Fig. S1). We therefore used sequential CRISPR-Cas9 to generate an HLA-A, B, and C triple knockout HeLa cell line (HeLa.ABC-KO) with almost no reactivity with W6/32 (supplemental Fig. S1). We then use a lentiviral construct to stably express HLA-B*51:01, the BD-associated HLA-B*51 subtype, in HLA.ABC-KO cells. This cell line is hereafter referred to as HeLa.ABC-KO.B51 (supplemental Fig. S2*A*). Notably the expression level of HLA class I was similar to that of primary peripheral blood human monocytes (supplemental Fig. S2*B*).

To study the effect of ERAP1 on the HLA-B*51:01 peptidome, we used a lentiviral shRNA to stably and efficiently (>90%) suppress endogenous ERAP1 expression in HeLa.ABC-KO.B51 cells (supplemental Fig. S2*C*).

##### Identification of An Unconventional HLA-B*51:01 Subpeptidome That has Neither Pro nor Ala at Position 2

**Fig. 1.**
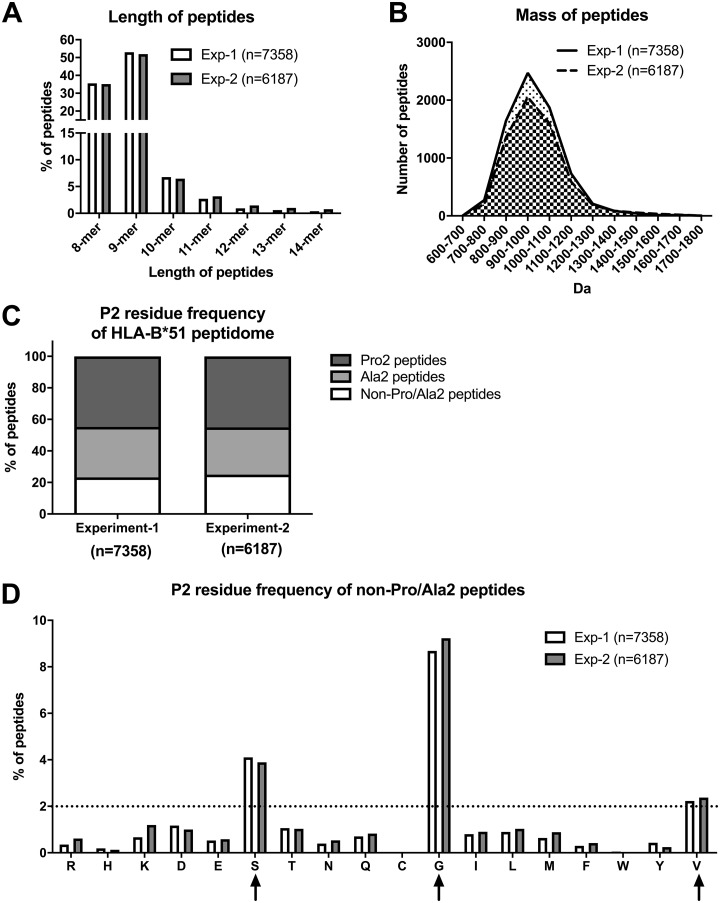
Identification of an unconventional HLA-B*51:01 subpeptidome that has neither proline nor alanine at position 2. Peptides bound to HLA-B*51 were eluted from HLA-B*51 complexes, purified from HeLa.ABC-KO.B51 cells using the W6/32 antibody. Length and mass of peptides identified are shown in (*A*) and (*B*) respectively. Amino acid usage of HLA-B*51 peptidome at position 2 is shown in (*C*) and (*D*). Two independent experiments are shown. The predominant residues (>2%) at P2 of non-Pro/Ala2 peptides were labeled using up arrow. Broken horizontal lines indicate 2% of total peptides in each group.

We first investigated the HLA-B*51:01-associated peptidome in the ERAP1-competent HeLa.ABC-KO.B51 cells. Cells were expanded to 1–5 × 10^8^, and peptides were eluted and sequenced from W6/32-immunopurified HLA-B*51:01 complexes. 7358 eluted HLA-B*51:01-bound peptides were sequenced. In a second independent experiment, we identified 6187 peptides, of which 4576 were common between two experiments. Very little difference in peptide length or mass distribution was found between the two experiments, highlighting the reproducibility and reliability of the data (Figs. 1*A* and 1*B*). We first focused on the amino acid usage at position 2 (P2), the key anchoring locus. In agreement with previous studies, Pro and Ala were predominant at position 2 (P2), with the frequencies of 45% and 30%, respectively (Fig. 1*C*) (13, 14). Interestingly, 24% of HLA-B*51:01-bound peptides did not have Pro or Ala at P2 (hereafter referred to as non-Pro/Ala2 peptides) (Fig. 1*C*), with glycine, serine, and valine accounting for 9%, 4%, and 2% of the total HLA-B*51:01 peptidome, respectively (Fig. 1*D*).

##### The non-Pro/Ala2 HLA-B*51:01 Subpeptidome has Characteristic Length Distribution and Residue Usage at P1 and PΩ

**Fig. 2.**
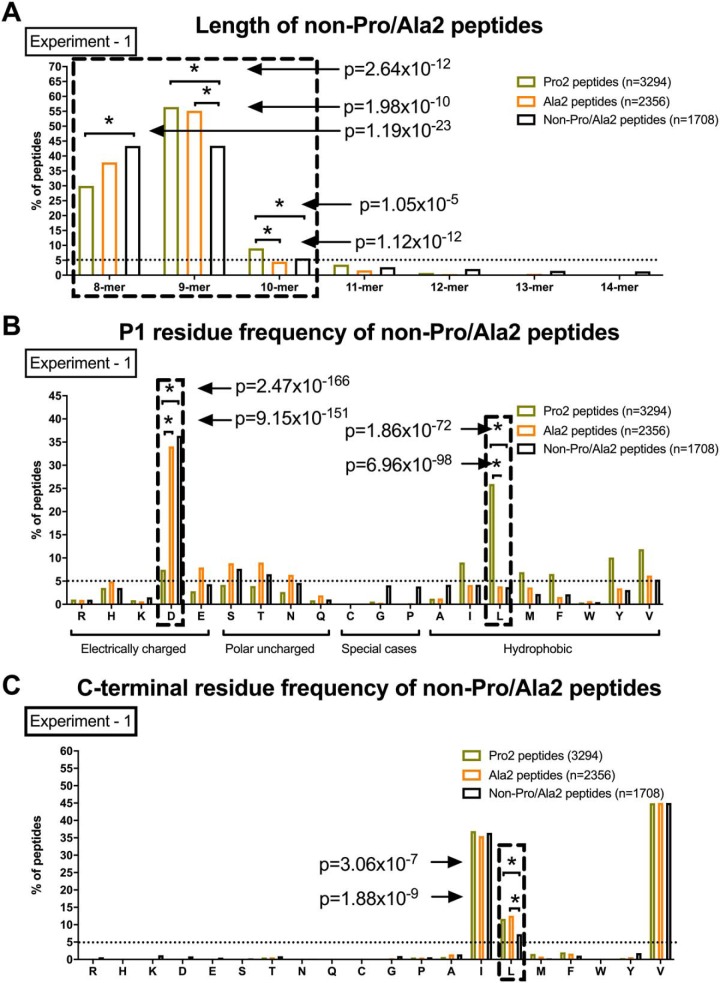
The non-Pro/Ala2 HLA-B*51:01 subpeptidome has characteristic length distribution and residue usage at P1 and PΩ. Length (*A*), P1 (*B*), and C-terminal (*C*) residue frequency of Pro2, Ala2 and non-Pro/Ala2 peptides are shown. One of two independent experiments is shown. Chi-squared test with Bonferroni correction used for statistics. Dashed box highlights the data with significant differences between Pro2, Ala2, and non-Pro/Ala2 peptides.

We next compared the length of non-Pro/Ala2 peptides with Pro2 and Ala2 subpeptidomes (Fig. 2*A* and supplemental Fig. S3*A*). Significantly more 8-mer and fewer 9-mer and 10-mer peptides were present in non-Pro/Ala2 peptides compared with the Pro2 subpeptidomes. Additionally, significantly fewer 9-mer peptides were present in non-Pro/Ala2 than Ala2 subpeptidomes. Interestingly, we also found that Pro2-peptides are longer than the other two peptide groups. This observation is likely explained by the low activity of ERAP1 against substrates carrying Pro at P2 (8), leading to an increased quantity of long Pro2-peptides supplied to HLA-B*51 in the ER.

The N-terminal (P1) residue usage of non-Pro/Ala2 peptides was very similar to Ala2 peptides, with aspartic acid as the dominant residues (∼30%, Fig. 2*B* and supplemental Fig. S3*B*). In contrast, the Pro2 subpeptidome had a significantly higher frequency of leucine at P1 than other two subpeptidomes, around 25% compared with 4 and 4%. While all three subpeptidomes had valine, isoleucine, and leucine as predominant residues at the C terminus (V > I > L), Leucine was significantly reduced in non-Pro/Ala2 peptides (Fig. 2*C* and supplemental Fig. S3*C*). Thus, non-Pro/Ala2 peptides comprise a significant subpeptidome that favors 8-mers and aspartic acid at N terminus.

##### ERAP1 Silencing Increases the Percentage of Long (10-mer and 11-mer) HLA-B*51:01-bound Peptides

**Fig. 3.**
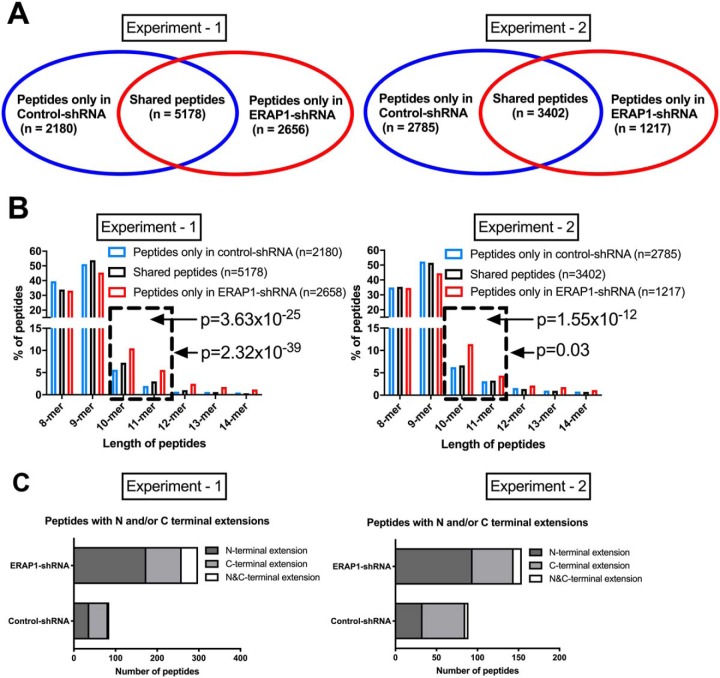
Silencing of ERAP1 leads to increased percentage of long (10-mer and 11-mer) HLA-B*51-bound peptides and generation of N and C-terminally extended peptides. Peptides bound to HLA-B*51 were eluted from HLA-B*51 complexes, purified from ERAP1-competent and -silenced HeLa.ABC-KO.B51 cells using the W6/32 antibody. Number and length distribution of peptides identified are shown in (*A*) and (*B*). Total peptide number and classification of peptides with N and/or C-terminal extensions are shown in (*C*). Two independent experiments were carried out. Chi-squared test with Bonferroni correction used for statistics in (*B*). Dashed box highlights long peptides (10-mer and 11-mer).

We next studied the effect of ERAP1 knockdown on the peptides bound to HLA-B*51:01. In experiment 1, 7358 and 7834 HLA-B*51-bound peptides were isolated from cells carrying control-shRNA and ERAP1-shRNA, respectively (Fig. 3*A*, *left-hand panel*). 5178 peptides were shared between the two cell lines. Slightly fewer peptides were identified in the second independent experiment (Fig. 3*A*, *left-hand panel*). Fig. 3*B* shows that the peptide pool unique to ERAP1-silenced cells has higher proportion of long peptides (10-mer and 11-mer) compared with the peptide pool unique to ERAP-competent cells (Fig. 3*B*). Interestingly, peptides shared between the two cell lines have similar length distribution to peptides that were only present in ERAP1-competent cells.

##### Both N- and C-terminally Extended Peptides can be Eluted from HLA-B*51:01 Following ERAP1 Silencing

As ERAP1 trims peptides from their N termini (8, 20), we hypothesized that N-terminally extended peptides would be enriched in ERAP1-silenced cells. Extended versions of peptides identified in either ERAP1-silenced or ERAP1-competent cells were identified (script used available upon request). In experiment 1, 298 extended peptides were identified in ERAP1-silenced cells, compared with only 85 in ERAP1-competent cells (Fig. 3*C*, *left-hand panel*). As expected, the majority of these peptides had N-terminal extensions (*n* = 174). Interestingly, 85 C-terminally extended and 39 N- and C-terminally extended peptides were also present. Similar results were found in experiment 2 (Fig. 3*C*, *right-hand panel*). Notably, we observed a unique pattern of P2 amino acid usage in N-terminally extended peptides from ERAP1-silenced HeLa.ABC-KO.B51 cells (supplemental Fig. S4). The dominance of Pro was lost, with a remarkable increase in frequency of aspartic acid to 20% from less than 2%.

##### ERAP1 Silencing Alters the Dominant Anchoring Amino Acid at Position 2 of HLA-B*51:01-bound Peptides

**Fig. 4.**
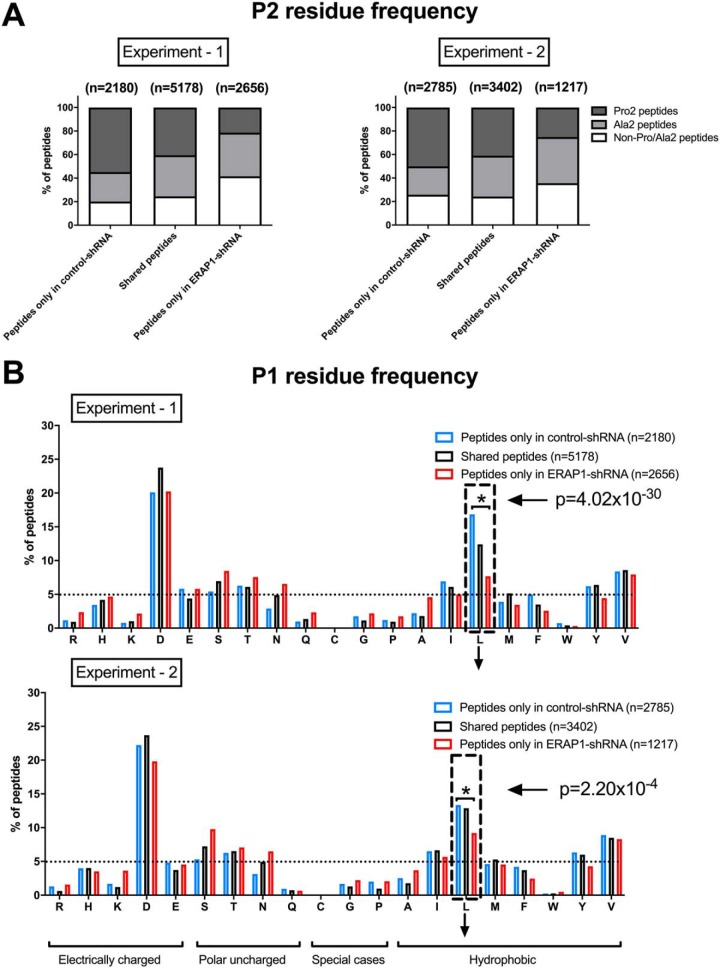
Silencing of ERAP1 alters amino acid usage at positions 1 and 2 of HLA-B*51-bound peptides, increasing the proportion of non-Pro Ala 2 residues at position 2. The percentage of HLA-B*51-bound peptides were assessed according to the abundance of amino acids at position 2 (*A*) and position 1 (*B*). The residues that were less abundant in ERAP1-silenced cells were labeled using down arrow. Two independent experiments were carried out. Chi-squared test with Bonferroni correction used for statistics. Dashed box highlights leucine. Broken horizontal lines indicate 5% of total peptides in each group.

We then investigated the effect of ERAP1 silencing on residue usage at position 2 (P2). In the peptides uniquely identified from cells carrying control-shRNA, we found 50–55% Pro2 peptides, 20–30% Ala2 peptides, and 20–25% non-Pro/Ala2 peptides (Fig. 4*A*). Knockdown of ERAP1 led to striking reduction in prevalence of Pro2 peptides to 20% and a significant increase in the frequency of non-Pro/Ala2 peptides (30–40%) (experiment 1: *p* = 2.90 × 10^−119^ and 4.08 × 10^−134^ for Pro2 and non-Pro/Ala2 peptides, respectively; experiment 2: *p* = 2.85 × 10^−34^ and 6.12 × 10^−11^ for Pro2 and non-Pro/Ala2 peptides, respectively). ERAP1 therefore has profound effects on the amino acid usage at P2 of HLA-B*51-bound peptides.

##### ERAP1 Silencing Reduces the Frequency of Leucine at Position 1 (P1)

We next examined the effect of ERAP1 on the position 1 (P1) amino acid frequencies of HLA-B*51-bound peptides. In ERAP1-competent cells, the dominant residues are aspartic acid and leucine, with the approximate frequencies of 20% and 15%, respectively (Fig. 4*B*, blue bars). ERAP1 silencing significantly reduced the frequency of leucine at P1 (*p* = 4.02 × 10^−30^ and 2.20 × 10^−4^ in experiments 1 and 2, respectively). The C-terminal residue usage was not affected (data not shown).

##### ERAP1 Silencing Increases the Length and Motif of HLA-B*51:01-bound Ala2-, non-Pro/Ala2- but not Pro2- Peptides

**Fig. 5.**
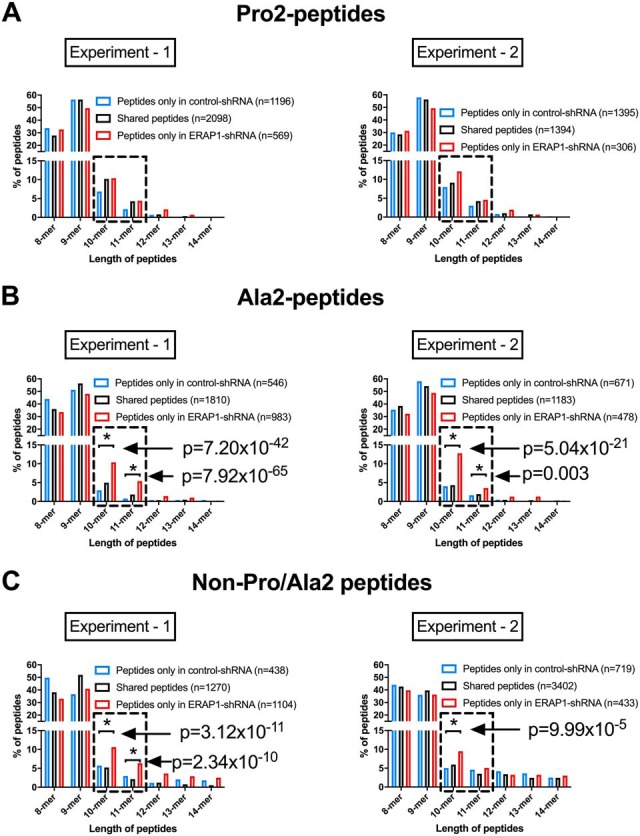
**Silencing of ERAP1 increases the length of Ala2 and non-Pro/Ala2 peptides but not Pro2 peptides.** Frequencies of peptides in different lengths were compared between ERAP1-competent and -silenced HeLa.ABC-KO.B51 cells within three subpeptidomes: Pro2 (*A*), Ala2 (*B*), and non-Pro/Ala2 (*C*) peptides. Two independent experiments were carried out. Chi-squared test with Bonferroni correction used for statistics. Dashed box highlights the data with significant differences between ERAP1-competent and -silenced cells.

**Fig. 6.**
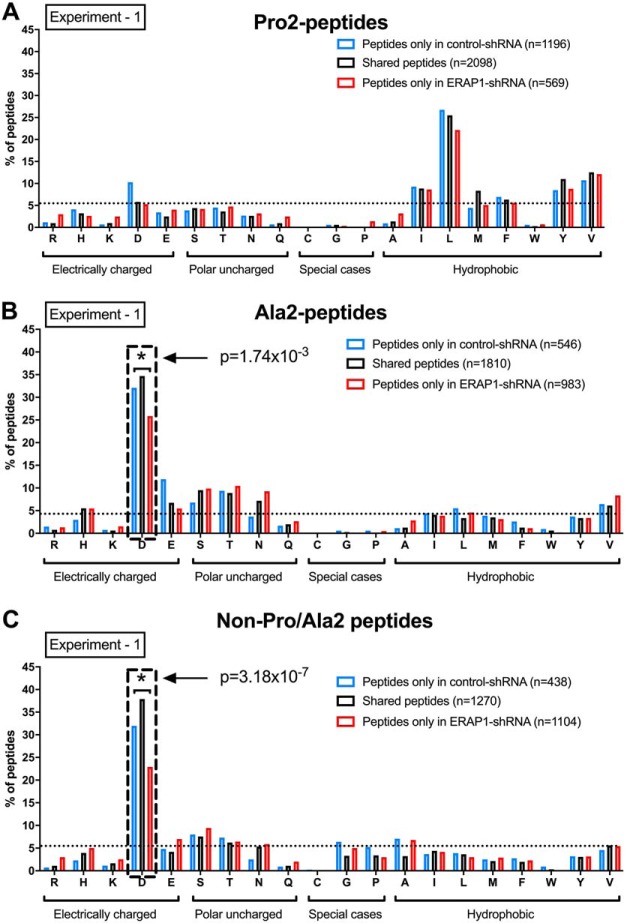
**Silencing of ERAP1 alters amino acid usage at positions 1 of Ala2 and non-Pro/Ala2 peptides but not Pro2 peptides.** Frequencies of peptides with different amino acids at position 1 were compared between ERAP1-competent and -silenced HeLa.ABC-KO.B51 cells within three subpeptidomes: Pro2 (*A*), Ala2 (*B*), and non-Pro/Ala2 (*C*) peptides. One of two independent experiments is shown. Chi-squared test with Bonferroni correction used for statistics. Dashed box highlights the data with significant differences between ERAP1-competent and -silenced cells.

Considering the differences in peptide length and motif between Pro2, Ala2, and non-Pro/Ala2 subpeptidomes, we hypothesized that their regulation by ERAP1 might be different. Silencing of ERAP1 had little effect on the length of Pro2-peptides (Fig. 5*A*). In contrast, increased percentages of 10-mer and 11-mer Ala2-peptides were present in ERAP1-silenced cells (Fig. 5*B*, *p* = 7.2 × 10^−42^ for 10-mer, *p* = 7.92 × 10^−65^ for 11-mer in experiment 1; *p* = 5.04 × 10^−21^ for 10-mer, *p* = 0.003 for 11-mer in experiment 2). The frequency of 10-mers in non-Pro/Ala2 peptides was also significantly increased in ERAP1-silenced cells (Fig. 5C, *p* = 3.12 × 10^−11^ in experiment 1, *p* = 9.99 × 10^−5^ in experiment 2). Moreover, ERAP1 knockdown significantly reduced the frequency of the predominant P1 residue (aspartic acid) in Ala2 and non-Pro/Ala2 peptide, without affecting the frequency of predominant P1 residue (leucine) in Pro2 peptides. (Fig. 6 and supplemental Fig. S5). Thus, ERAP1 silencing affects the peptide length and motif of Ala2 and non-Pro/Ala2 peptides, but not Pro2 peptides.

##### ERAP1 Silencing Affects the Expression of Cell Surface HLA-B*51:01 in a Cell Type - Dependent Manner

**Fig. 7.**
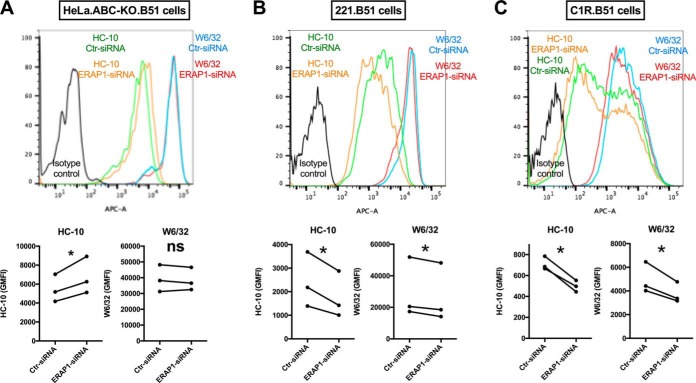
ERAP1 regulates HLA-B*51 cell surface expression in a cell-type-dependent manner. The effect of ERAP1 knockdown on HLA-B*51 expression is shown in three cell lines: HeLa.ABC-KO.B51 (*A*), 221.B51 (*B*), and C1R.B51 (*C*). HLA-B*51 β2m-FHC (HC-10) and classical HLA-B*51 complex (W6/32) cell surface expression is shown. Representative FACS plots are shown. *p* value was determined using unpaired two-tailed *t*-test (* = *p* < 0.05). All experiments were repeated three times.

ERAP1 is known to regulate MHC class I cell surface expression, we have shown a reduction of HLA-B*27 β2-microglobulin (β2m)-free heavy chain (FHC) cell surface expression following ERAP1 silencing or inhibition (8, 12). We therefore investigated the effects of ERAP1 on HLA-B*51 expression (12). We found that HLA-B*51 FHCs, stained by HC-10, were increased by knockdown of ERAP1 in HeLa.ABC-KO.B51 cells (Fig. 7*A*). We then studied two human B lymphoblastoid cell lines, 221.B51 and C1R.B51, and found reduced FHCs following ERAP1 silencing (Figs. 7*B* and 7*C*). Notably, cell surface expression of classical HLA-B*51 was not affected by ERAP1 silencing in HeLa.ABC-KO.B51 cells, but overall HLA class I levels were reduced in 221.B51 and C1R.B51 cells. These distinct results between epithelial (HeLa) and B (221 and C1R) cell lines suggest that ERAP1 has cell-type-dependent effects on HLA-B*51:01 expression.

## DISCUSSION

ERAP1 polymorphisms have recently been shown to affect the risk of developing BD in HLA-B*51 positive individuals (2, 3). Here we identify a previously understudied subpeptidome of HLA-B*51 that does not carry the classic Pro or Ala anchoring residues at position 2 (P2). This unconventional non-Pro/Ala2 subpeptidome accounts for more than 20% of peptides eluted from HLA-B*51 and has unique length distribution and peptide motif. Importantly, silencing of ERAP1 substantially increased the percentage of non-Pro/Ala2 peptides and of long (10-mer and 11-mer) peptides bound to HLA-B*51. Following ERAP1 silencing extended peptides could be detected, with extensions predominantly but not exclusively at the N termini. Interestingly knockdown of ERAP1 altered the length and N-terminal residue usage of non-Pro/Ala2 and Ala2 but not the Pro2 peptides. Last, cell surface expression of HLA-B*51 β2m-FHCs was also regulated by ERAP1. Our results provide possible explanations for the genetic association of ERAP1 with BD through alteration of HLA-B*51 peptide presentation and cell surface FHC expression.

Lack of a selective antibody for HLA-B*51 has been a significant obstacle in studying the HLA-B*51 peptidome. Thus, W6/32, the antibody previously used to isolate HLA-B*51 complexes (14, 15), binds most other HLA class I alleles in addition to HLA-B*51. This is an important consideration even in studies using HLA class I-low cells such as 721.221 and C1R cells, and previous studies therefore concentrated on classical Pro2 or Ala2 HLA-B*51 peptidomes. We here describe a novel HLA-A, B, and C CRISPR-Cas9 knockout HeLa cell line, facilitating the isolation of high purity HLA-B*51 complexes in this study, which allows us to investigate the unconventional HLA-B*51 subpeptidome that lack classic P2 anchoring residues Pro and Ala. This cell line also provides a tool for the future study of other HLA class I molecules. In addition, we identified more than 4000 peptides in each cell line studied. The high number of HLA-B*51-bound peptides eluted allows us to perform detailed peptide length and sequence analysis in subgroups of HLA-B*51 peptidome, such as non-Pro/Ala2, Pro2-, and Ala2-peptides.

Peptide contamination is a potential problem in any HLA peptidome study and cannot be formally excluded in the present study. However, the non-Pro/Ala2 HLA-B*51 subpeptidome identified in this study is unlikely due to contamination for the following reasons. First, the non-Pro/Ala2 subpeptidome identified mainly comprises 8-mers (40–45%) and 9-mers (35–40%), consistent with the length features of HLA class I peptidomes. Second, the non-Pro/Ala2 subpeptidome is characterized by amino acid usage at P1 and P Ω that is very similar to the Ala2 subpeptidome already described for HLA-B*51 (and confirmed here). Previous studies have identified non-Pro/Ala2 peptides (10% in 14) but either did not comment in detail or filtered them out from analysis (15), presumably because they could not exclude contamination since they lacked an HLA-B*51-specific antibody and studied cell lines expressing multiple HLA class I alleles. Last, the NetMHCpan4.0 in silico peptide binding algorithm predicts that 70% of 9-mers and 50% of 10-mers from the non-Pro/Ala2 peptidome bind to HLA-B*51 (supplemental Fig. S6). Considering the bias of the algorithm toward Pro2 and Ala2 peptides, these findings strongly support that these non-Pro/Ala2 peptides are indeed ligands for HLA-B*51.

We have also performed correlation analysis using the intensities of peptides (as a measure of abundance) that are shared between different conditions (quantitative search performed using Peak 8.5). A stronger correlation was present between biological replicates (Exp1 Ctr-shRNA and Exp2 Ctr-shRNA or Exp 1 ERAP1-shRNA and Exp2 ERAP1-shRNA) than between Ctr-shRNA and ERAP1-shRNA cells (supplemental Fig. S7). Interestingly, ERAP1 knockdown increases the abundance of non-Pro/Ala2 peptides, Ala2 peptides, and 10-mer peptides, in agreement with the findings from nonshared peptides.

Pro2 peptides cannot be transported into ER by TAP (21), therefore, were either generated in the ER or transported into the ER through TAP-independent mechanism. ERAP1 does not trim Pro2 peptides and thus could have a major role in generating ER Pro2 peptides. In line with this, we found that ERAP1 knockdown significantly reduced the abundance of Pro2 HLA-B*51 binding peptides. Notably, in the ERAP1-silenced cells, Pro2 peptides still made up 20–30% of HLA-B*51 ligands, supporting the TAP-independent transportation or generation of Pro2 peptides in the ER. Indeed, Pro2 HLA-B*51 peptide ligands have been found to be present in both TAP-deficient LCL721.174 and its TAP-expressing progenitor cell lines (22).

Interestingly, we found that knockdown of ERAP1 increased the length of non-Pro2 (non-Pro/Ala2 and Ala2) but not the Pro2 peptides. A key difference between Pro2 and non-Pro2 peptides is that the Pro2 peptides cannot be cleaved by ERAP1 (9). Thus, it is likely that long Ala2 but not long Pro2 peptides have been “destroyed” by ERAP1 in normal cells, resulting in the accumulation of long Pro2 peptides with in the ER. This assumption is supported by the increased frequency of long peptides (mainly 10-mer) in Pro2 subpeptidome compared with Ala2 or non-Pro/Ala2 subpeptidomes (Fig. 2*A* and Fig. S3*A*). Knockdown of ERAP1 spares the long non-Pro2 peptides from being cleaved thus increases their abundance in the ER but would not do so for the long Pro2 peptides. In other words, more long non-Pro2 peptides but not long Pro2 peptides are supplied to HLA-B*51 when ERAP1 is silenced, resulting in the different effects of ERAP1 knockdown on the length of Pro2 and non-Pro2 peptides.

We here also show that, at position 1 (P1), ERAP1 knockdown unexpectedly reduces the frequency of leucine, a residue highly susceptible to ERAP1 enzymatic cleavage. Through analysis of P1 residue frequency of non-Pro/Ala2, Pro2-, and Ala2-peptides, we demonstrate that this finding is due to the predominance of leucine at P1 of Pro2-peptides, which are hugely reduced by knockdown of ERAP1.

Although ERAP1 silencing resulted in generation of (mainly N-terminally) extended peptides, peptides with C-terminal, and both N- and C-terminal extensions were also identified. These C-terminally extended peptides are unlikely to be contaminants because, first, they share similar amino acid usage at P1, P2, and P Ω with HLA-B*51 peptides eluted from ERAP1-competant cells (supplemental Fig. S8*B–D*). Second, more than 50% of these C-terminally extended peptides are predicted to bind to HLA-B*51 by NetMHCpan 4.0 (supplemental Fig. S9). These findings strongly support that they are indeed ligands for HLA-B*51, despite their increased length. But why has knockdown of ERAP1, an N-terminal trimmer, resulted in generation of C-terminally extended peptides? For the majority of the C-terminal extended peptides eluted from ERAP1-silenced cells (78 out of 85 for experiment 1, 40 out of 50 for experiment 2), their “root” peptides are present both wild-type and ERAP1-knockdown cells. Thus, it is likely that these C-terminal extended peptides, albeit being able to bind to HLA-B*51, are destroyed by ERAP1 in wildtype cells. In the absence of ERAP1, these peptides could be spared from digestion and could therefore have the chance to bind to HLA-B*51. For example, if both of ZXXXXXXXX (9-mer) and ZXXXXXXXXYY (11-mer) bind to HLA-*51, and ZXXXXXXXXYY (11-mer) was digested to XXXXXXXYY (a 9-mer that does not bind to HLA-B*51) by ERAP1, only ZXXXXXXXX (9-mer) would be detected in wild-type cells. In the absence of ERAP1, both ZXXXXXXXX (9-mer) and ZXXXXXXXXYY (11-mer) would be preserved and presented by HLA-B*51. However, this assumption does not explain the presence of C-terminally extended peptides are Pro2-peptides (around 20%), which cannot be cleaved by ERAP1. Thus, the shifting repertoire of peptides in the ER due to abrogated ERAP1 function could also contribute to the generation of the C-terminal extended peptides.

We have shown previously that most extended peptides bound to HLA-B*27 following ERAP1-silencing have C-terminal extensions (10). A possible explanation for this difference between HLA-B*51 and HLA-B*27 is their different stringency for position 2 (P2) anchor residues. More than 90% of HLA-B*27-bound peptides carry arginine at P2 (23), whereas HLA-B*51 is more permissive and harbors Pro and Ala, as well as other residues at P2. In line with this, we found that the predominance of Pro and Ala at P2 disappeared in N-terminally extended peptides from ERAP1-silenced HeLa.ABC-KO.B51 cells.

An ERAP1 coding variant, Arg725Gln, confers risk to BD but is protective for ankylosing spondylitis (2, 4). This variant exhibits lower enzyme activity than wild-type ERAP1 in trimming of N-terminal tryptophan from 10-mer peptide WRVYEKCALK, suggesting that it is likely a loss-of-function variant (4). However, additional substrates have not been tested, and, given that it is now widely accepted that ERAP1 activity is substrate dependent, we do not know yet if this BD-associated ERAP1 variant is underactive for all peptides.

The impact of ERAP1 on the HLA-B*51 peptidome has been studied previously by Guasp and colleagues comparing two B cell lines: HLA-B*51:08-expressing BCH-30 and HLA-B*51:01-expressing 721.221 cells (15). They found longer peptides and fewer Pro2-peptides in the cell line expressing a loss-of-function ERAP1 variant. Our study supports these findings but additionally identifies a significant non-Pro/Ala2 HLA-B*51 subpeptidome, markedly increased in abundance following ERAP1 silencing, that their methodology could not identify. Guasp and colleagues applied a peptide filter using P2 and PΩ (P9) anchor residues, in part because the BCH-30 cell line expressed multiple W6/32-reactive HLA molecules (including HLA-A*02:01, HLA-B*40:01, C*16:02, and C*16:03). By using the HLA-B*51:01-expressing HLA-A, B, and C knockout HeLa cells, the present study avoids these sources of variation, although the presence of only a single HLA class 1 molecule in our study could potentially have introduced bias due to the lack of other competing HLA molecules.

Notably, knockdown of ERAP1 increased the expression of cell surface HLA-B*51 β2m-FHCs in HeLa.ABC-KO.B51 cells but exhibited opposite effects in two B cell lines, 221.B51 and C1RB51. This suggests that the effect of ERAP1 on HLA-B*51 might be cell-type-dependent with potential differences between “professional” antigen-presenting cells such as B cells and epithelial cells such as HeLa cells. An alternative but less likely explanation is that the opposite effects between HeLa cells and B cells were due to ERAP2 differences. HeLa, unlike 221 and C1R cells, do not have endogenous ERAP2 (10). Although ERAP2 is not genetically associated with BD and is absent in around 25% of the population (24), it has been shown to trim peptides in concert with ERAP1 (25). However, our previous work shows that knockdown of ERAP1 had similar effects on cell surface HLA-B*27 FHC expression in ERAP2-deficient HeLa.B27 and ERAP2-competent C1R.B27 cells (12), and it has been shown in human primary cells that the absence of ERAP2 does not alter cell surface expression of HLA-B*27 (24).

The increased level of HLA-B*51 β2m-FHCs following ERAP1 knockdown in HeLa.ABC-KO.B51 cells is most likely due to the reduced stability of HLA-B*51 complexes. This assumption is supported by the reduced HLA-B*51-binding affinity of peptides eluted from ERAP1-silenced cells (supplemental Fig. S10). In line with this, Pro2 HLA-B*51 peptide ligands, which are less frequent in ERAP1-silenced cells, have been shown to bind HLA-B*51 better than Ala2 peptides (14).

Glycine is known as a position 2 anchor residue for HLA-B*51-binding peptides, albeit weaker than Pro2 and Ala2 (26). The non-Pro/Ala/Gly2 peptides make up only around 15% of all peptides eluted, but share similar peptide length and motif (P1 and PΩ amino acid usages) with the later (supplemental Fig. S11). Thus, these peptides are unlikely contaminants.

Several cell types have been implicated in the pathogenesis of BD, including cytotoxic CD8+ T cells, NK cells, and Th17 cells (reviewed in (6, 7, 27)). We here show that knockdown of ERAP1, which mimics the loss-of-function ERAP1 variants associated with the risk of developing BD, causes the substitution of 30–40% of HLA-B*51-bound peptides by new peptides. This is associated with a reduced frequency of Pro2-peptides and an increased frequency of non-Pro/Ala2-peptides. These findings support the idea that ERAP1 could possibly facilitate or enhance the presentation of immunogenic peptides in BD. We also show that knockdown of ERAP1 in HeLa.ABC-KO.B51 cells leads to increased length of HLA-B*51-bound peptides including N- (and C-) terminally extended peptides, which likely have low affinity to HLA-B*51. Indeed, in HeLa.ABC-KO.B51 cells, ERAP1 silencing increased the level of HLA-B*51 β2m-FHCs. Therefore, alteration of HLA-B*51 stability and expression by ERAP1 is another potential pathogenic mechanism in BD. Altered cell surface HLA-B*51 expression may have immunological effects through interaction with killer-cell immunoglobulin-like receptors or leukocyte immunoglobulin-like receptors expressed on immune cells.

Overall, we have used novel methodology to identify an unconventional but surprisingly abundant non-Pro/Ala2 HLA-B*51:01 subpeptidome, whose abundance is further substantially increased by silencing of ERAP1, a gene also strongly associated with BD. We additionally show that ERAP1 regulates HLA-B*51 cell surface expression in cell-type-dependent manner. These findings provide insights for the pathogenic roles of ERAP1 and HLA-B*51 in BD.

## DATA AVAILABILITY

The mass spectrometry proteomics data have been deposited to the ProteomeXchange Consortium via the PRIDE partner repository with the dataset identifier PXD013064. Sequences source and abundance of peptides eluted are shown in supplemental Tables 1 and 2.

## Supplementary Material

Supplementary figure 1-11

Supplementary table 1

Supplementary table 2

## Footnotes

^1^ The abbreviations used are:

BD: Behçet's disease
ERAP1: endoplasmic reticulum aminopeptidase 1
ER: endoplasmic reticulum
MHC: major histocompatibility complex
Pro: proline
FHC: free heavy chains
Ala: alanine
CRISPR: clustered regularly interspaced short palindromic repeats
TAP: transporter associated with antigen processing.
