# Supplementary material for: Identification of an Unconventional Subpeptidome Bound to the Behçet's Disease-associated HLA-B*51:01 that is Regulated by Endoplasmic Reticulum Aminopeptidase 1 (ERAP1)
Source: Mol Cell Proteomics. 2020 Mar 11;19(5):871–83. doi: 10.1074/mcp.RA119.001617 (PMC7196583; doi:10.1074/mcp.RA119.001617)
Supplement: Supplementary figure 1-11 [file 153410_2_supp_428124_q0v91j.pdf]

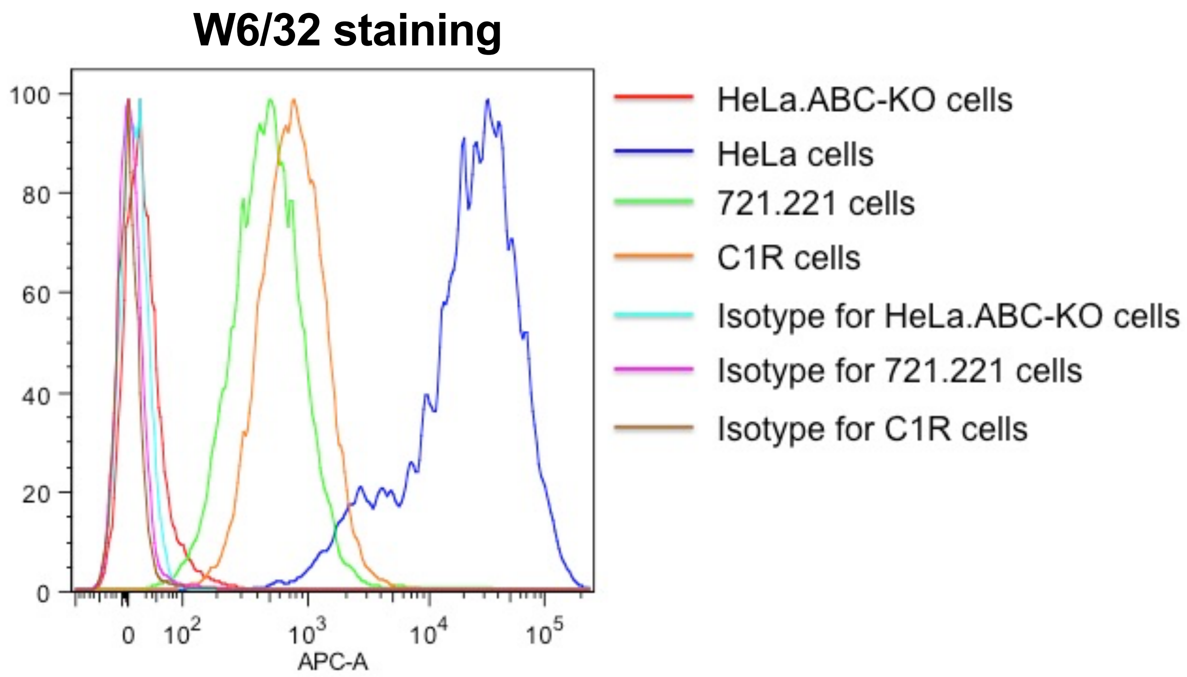

Figure S1. Knockout of HLA-A, B and C in HeLa cells largely abrogates reactivity with W6/32. W6/32 antibody was used to stain HeLa.ABC-KO, HeLa, 721.221 and C1R cells. Isotype control antibody staining was carried out. Experiments were repeated twice, representative FACS plot is shown.

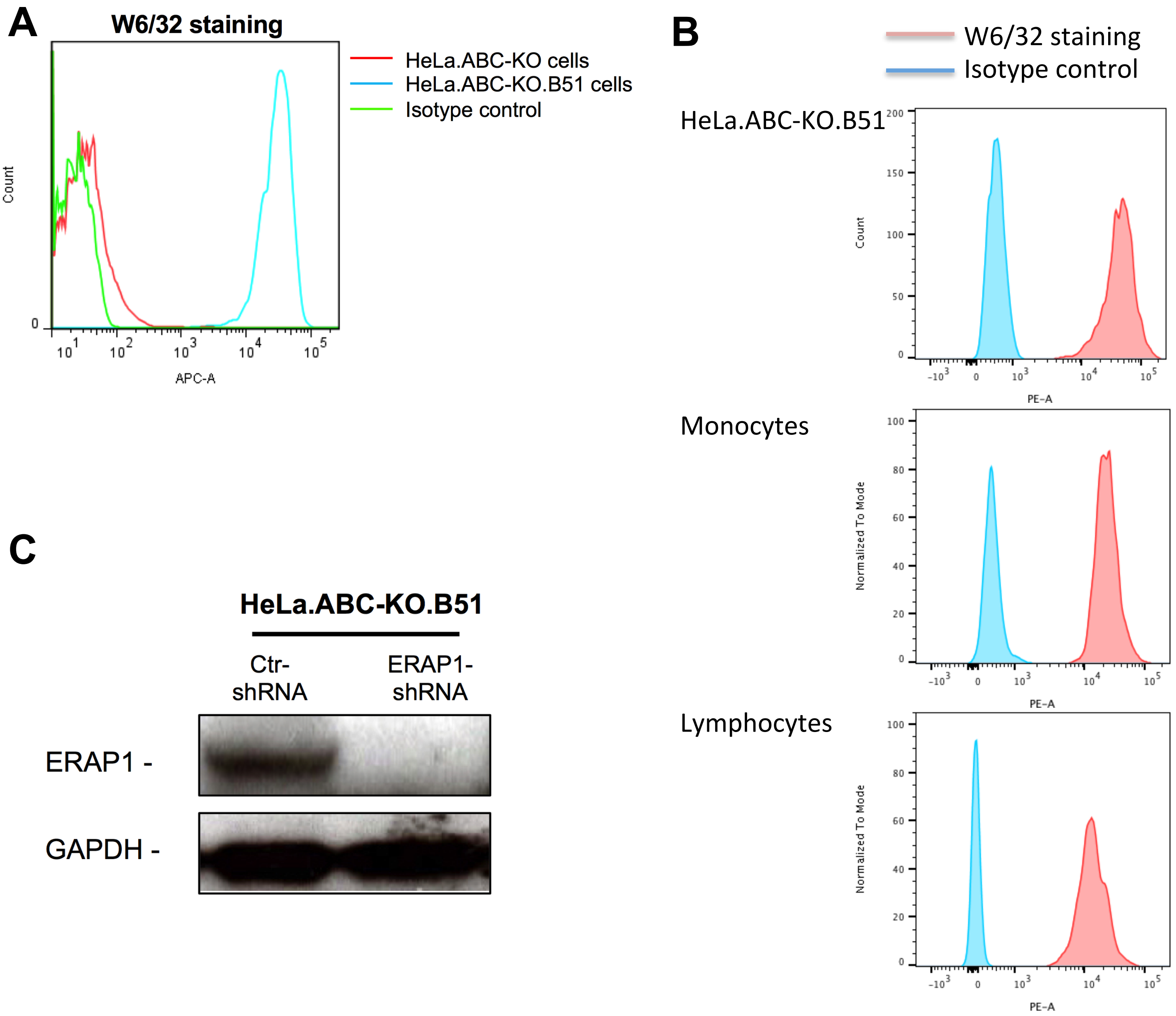

Figure S2. Expression of HLA-B*51 in HeLa.ABC-KO cells and stable silencing of ERAP1 in HLA.ABC-KO.B51 cells. (A) W6/32 antibody was used to stain HeLa.ABC-KO and HeLa.ABC-KO.B51 cells. Isotype control staining was carried out using HeLa.ABC-KO cells. (B) W6/32 and isotype control antibody have been used to measure the cell surface HLA class I expression levels in HeLa.ABC-KO.B51 cells, primary monocytes and lymphocytes. (C) Western blot showing efficient silencing of endogenous ERAP1 in HeLa.ABC-KO.B51 cells.

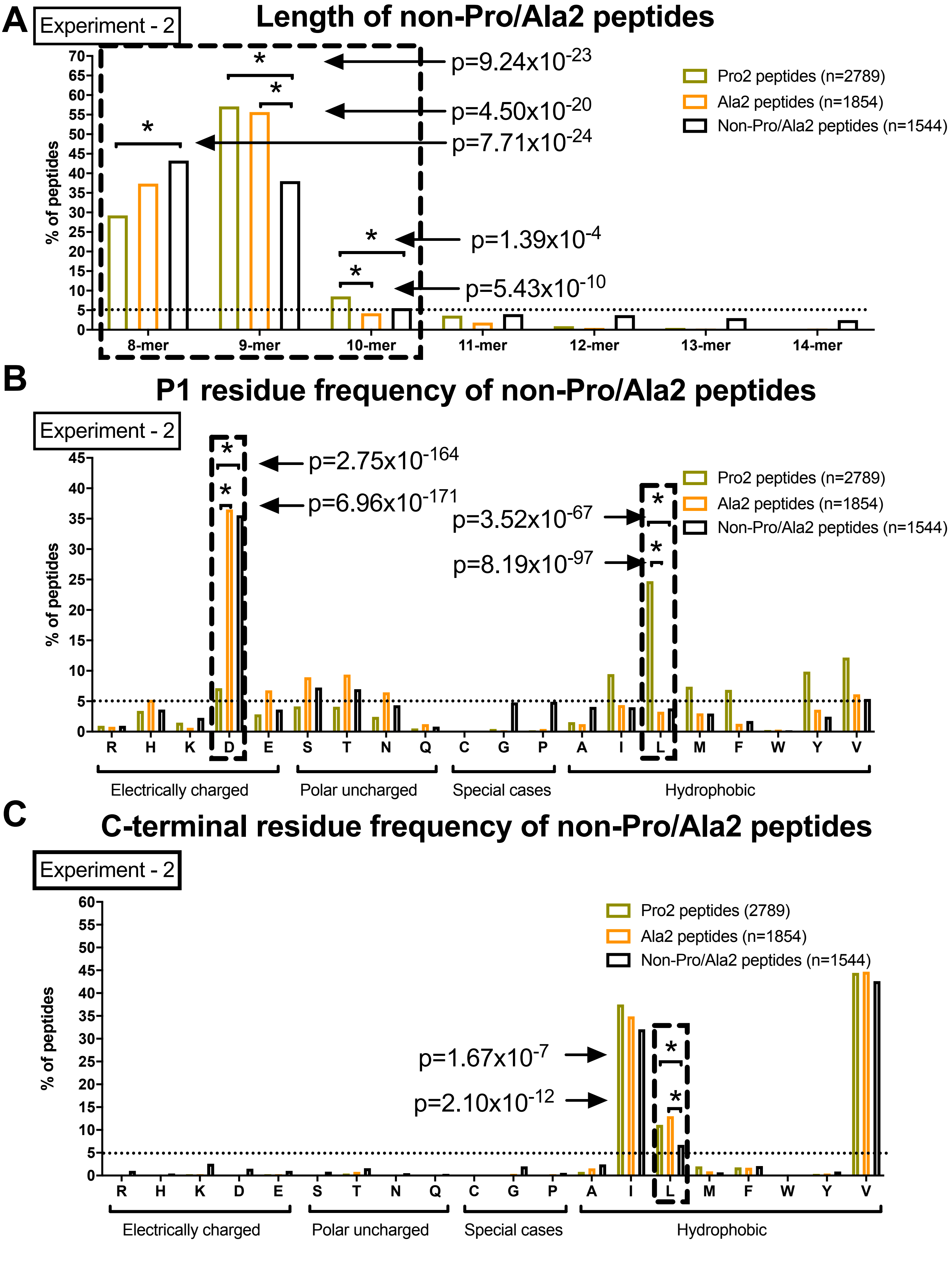

Figure S3. The non-Pro/Ala2 HLA-B*51:01 sub-peptidome has characteristic length distribution and residue usage at P1 and PΩ. Length (A), P1 (B) and C-terminal (C) residue frequency of Pro2, Ala2 and non-Pro/Ala2 peptides are shown. One of two independent experiments is shown. Chi-squared test with Bonferroni correction used for statistics.

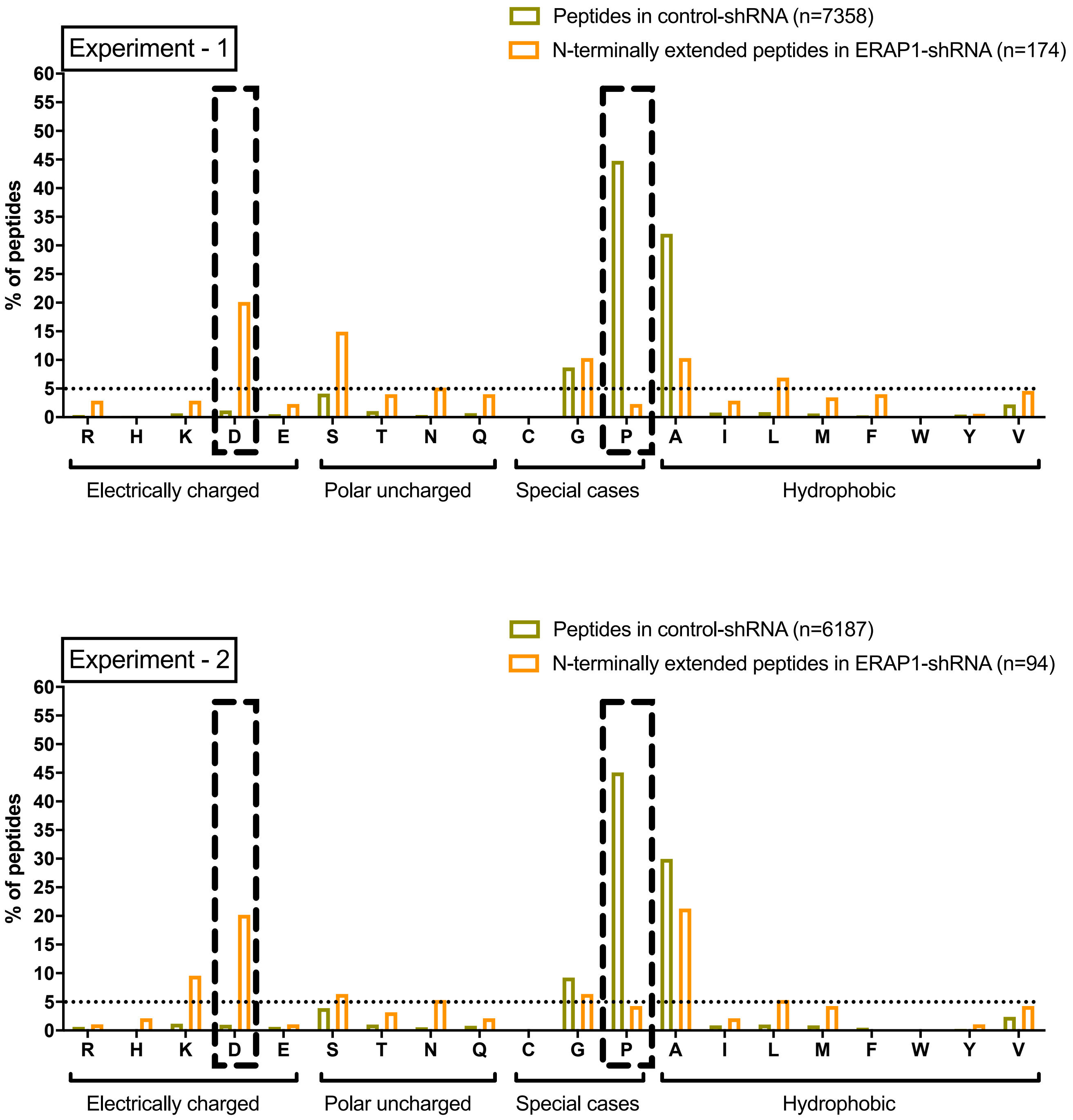

Figure S4. The predominance of Proline at position 2 is absent in N-terminally extended peptides found in ERAP1-silenced cells. Frequencies of amino acids at position 2 was compared between peptides present in ERAP1-competent cells and N-terminally extended peptides present in ERAP1-silenced cells. Two independent experiments were carried out.

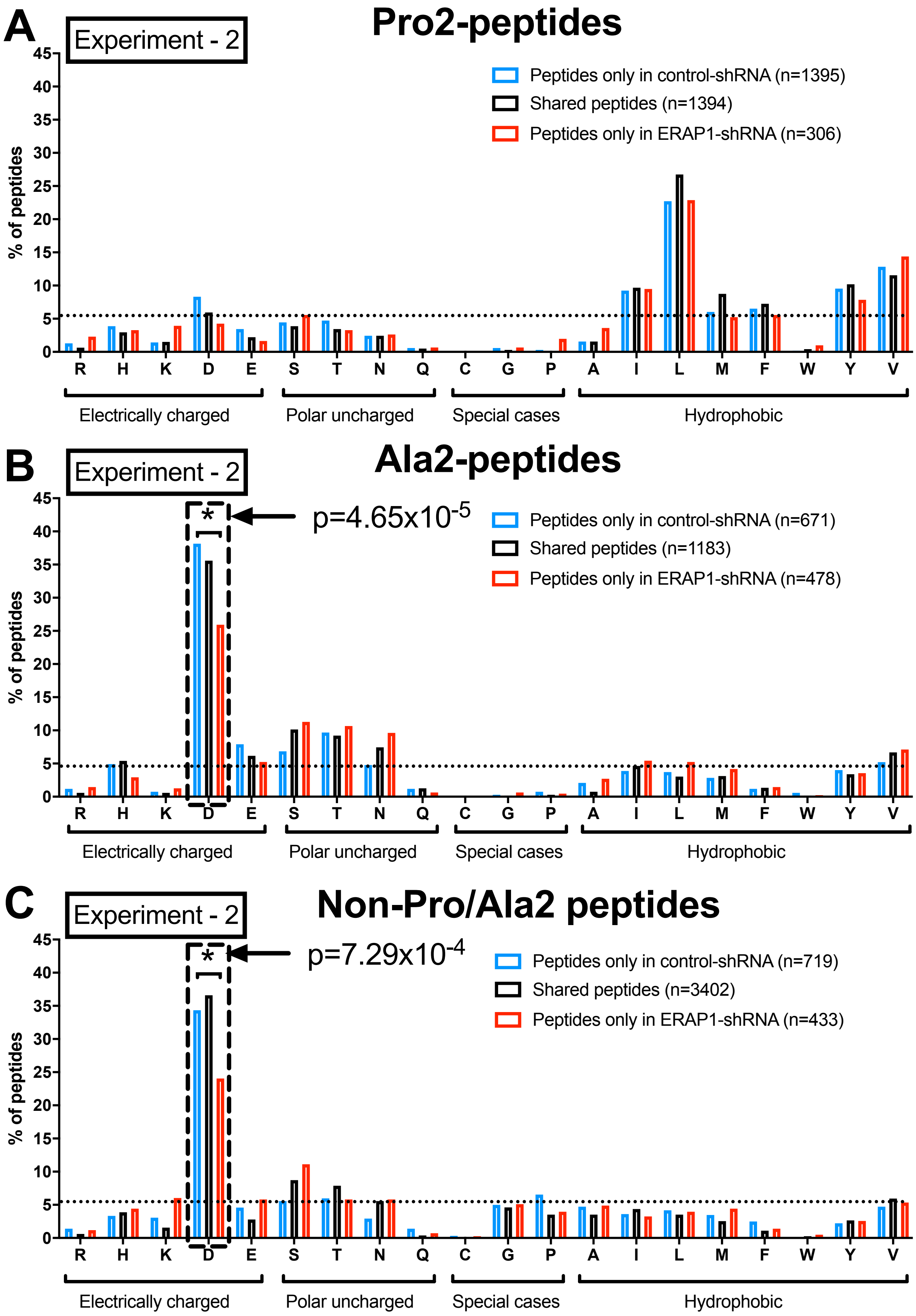

Figure S5. Silencing of ERAP1 alters amino acid usage at positions 1 of Ala2 and non-Pro/Ala2 peptides but not Pro2 peptides. Frequencies of peptides with different amino acids at position 1 were compared between ERAP1-competent and-silenced HeLa.ABC-KO.B51 cells within three sub-peptidomes: Pro2 (A), Ala2 (B) and non-Pro/Ala2 (C) peptides. One of two independent experiments is shown. Chi-squared test with Bonferroni correction used for statistics.

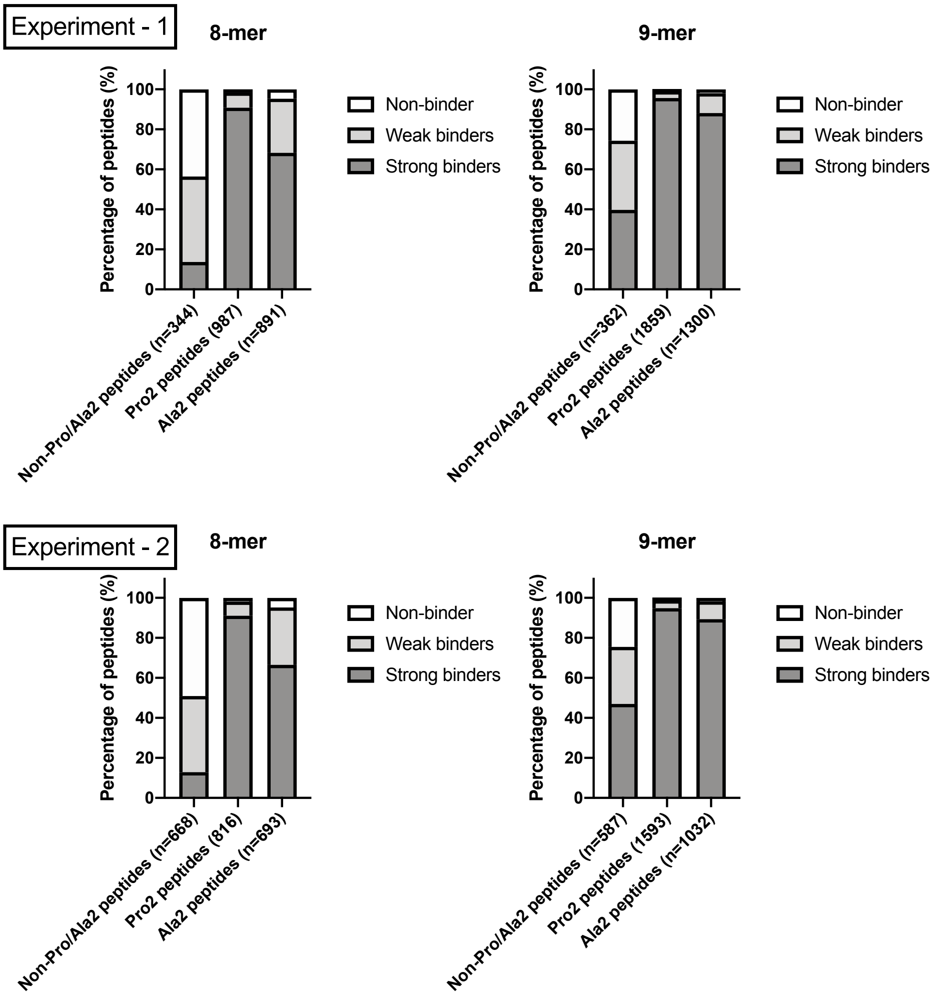

Figure S6. Predicted HLA-B*51:01 binding affiniy of eluted peptides. NetMHCpan4.0 was used for in silico prediction

A

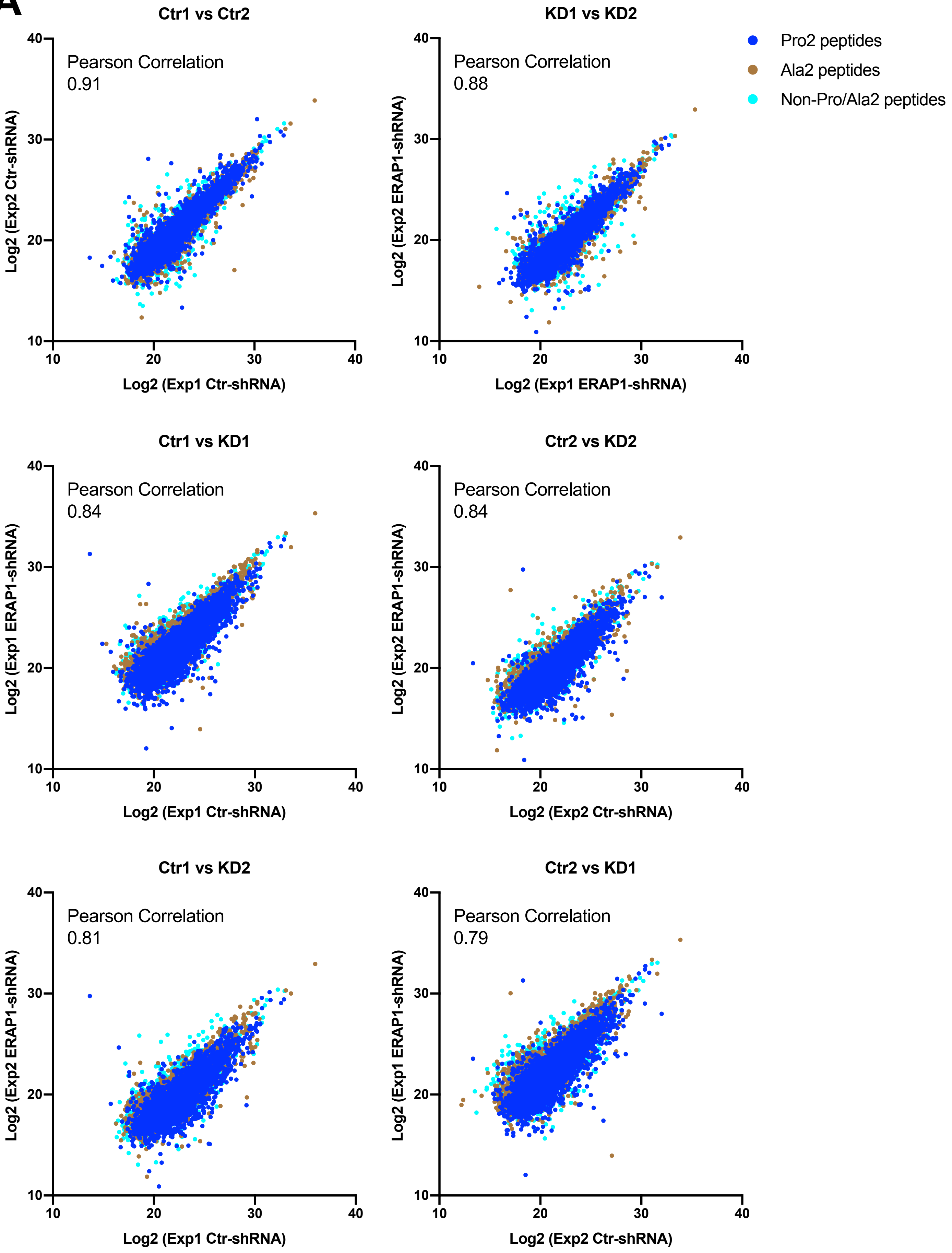
Ctr1 vs Ctr2
Pearson Correlation 0.91
Log2 (Exp2 Ctr-shRNA)
Log2 (Exp1 Ctr-shRNA)
KD1 vs KD2
Pearson Correlation 0.88
Log2 (Exp2 ERAP1-shRNA)
Log2 (Exp1 ERAP1-shRNA)
Pro2 peptides
Ala2 peptides
Non-Pro/Ala2 peptides
Ctr1 vs KD1
Pearson Correlation 0.84
Log2 (Exp1 ERAP1-shRNA)
Log2 (Exp1 Ctr-shRNA)
Ctr2 vs KD2
Pearson Correlation 0.84
Log2 (Exp2 ERAP1-shRNA)
Log2 (Exp2 Ctr-shRNA)
Ctr1 vs KD2
Pearson Correlation 0.81
Log2 (Exp2 ERAP1-shRNA)
Log2 (Exp1 Ctr-shRNA)
Ctr2 vs KD1
Pearson Correlation 0.79
Log2 (Exp1 ERAP1-shRNA)
Log2 (Exp2 Ctr-shRNA)

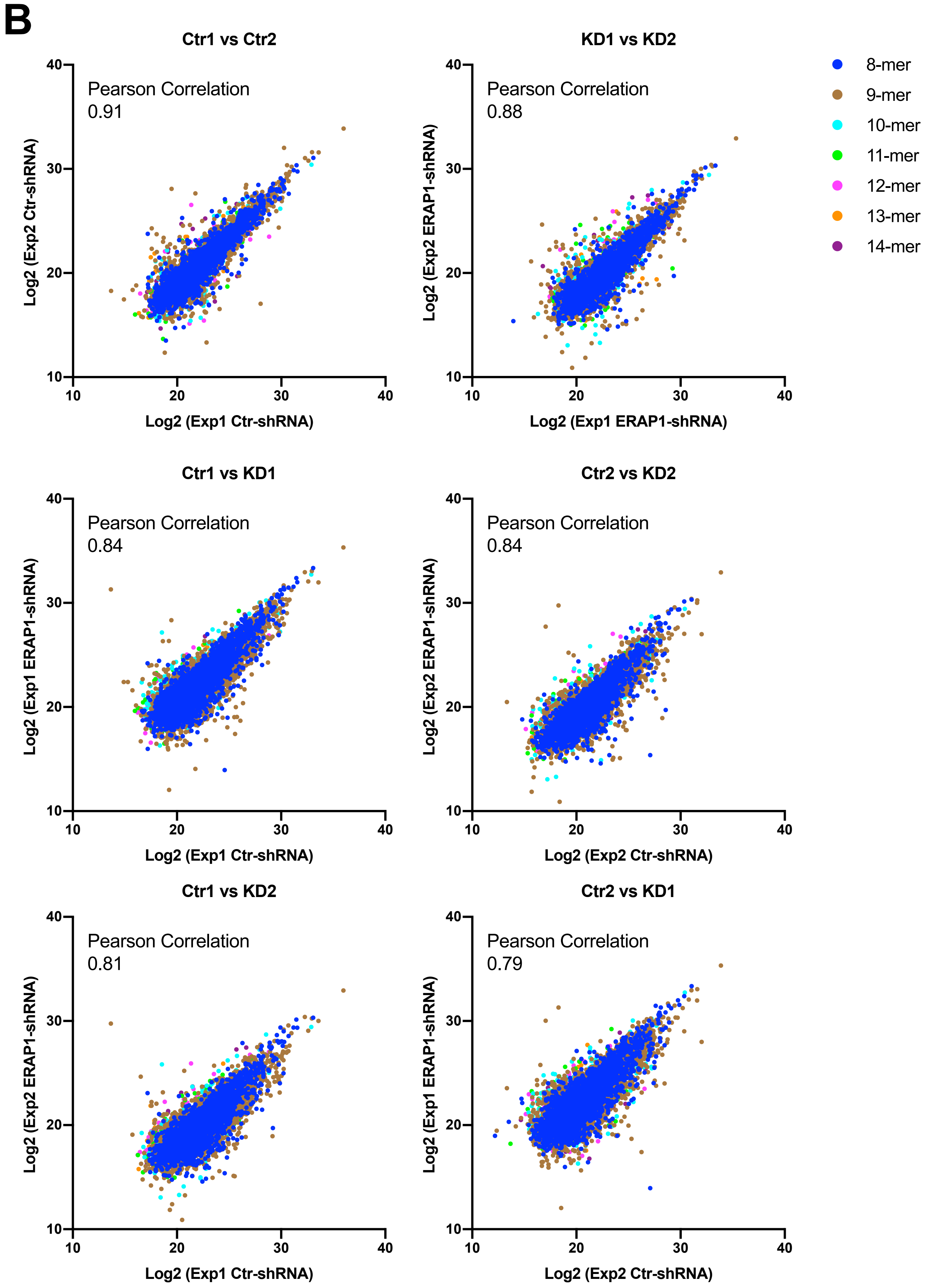

Figure S7. Correlation in intensities of peptides shared between Ctr-shRNA and ERAP1-shRNA from Exp1 and Exp2. The pearson correlation analysis has been perfromed. Pro2, Ala2 and Non-Pro/Ala2 peptides have been color-coded in (A). Peptides with different lengths have been color-coded in (B).

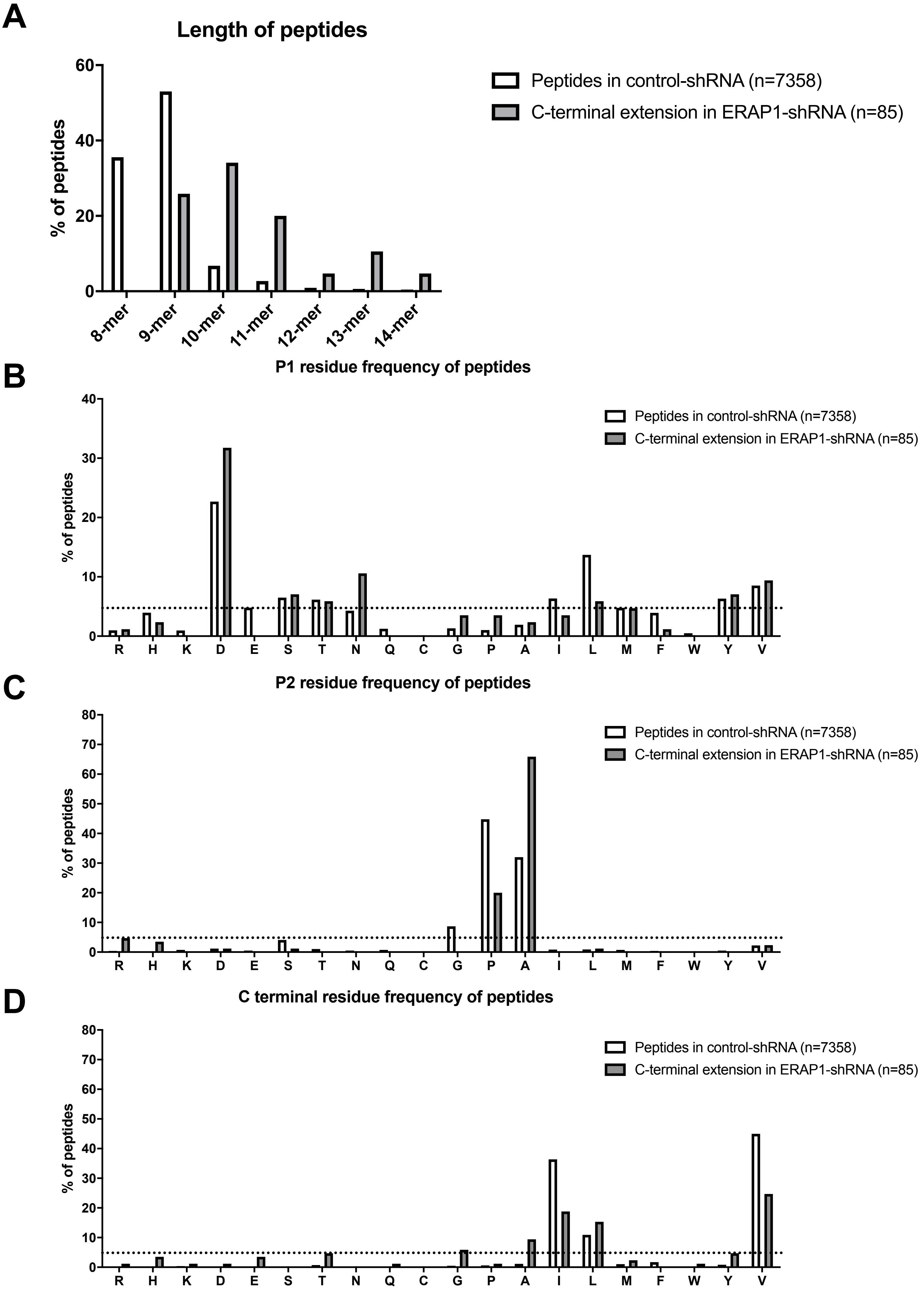

Figure S8. Length and peptide motif of C-terminally extended peptides in ERAP1-silenced cells. Peptides eluted in Exp-1 was used for analysis.

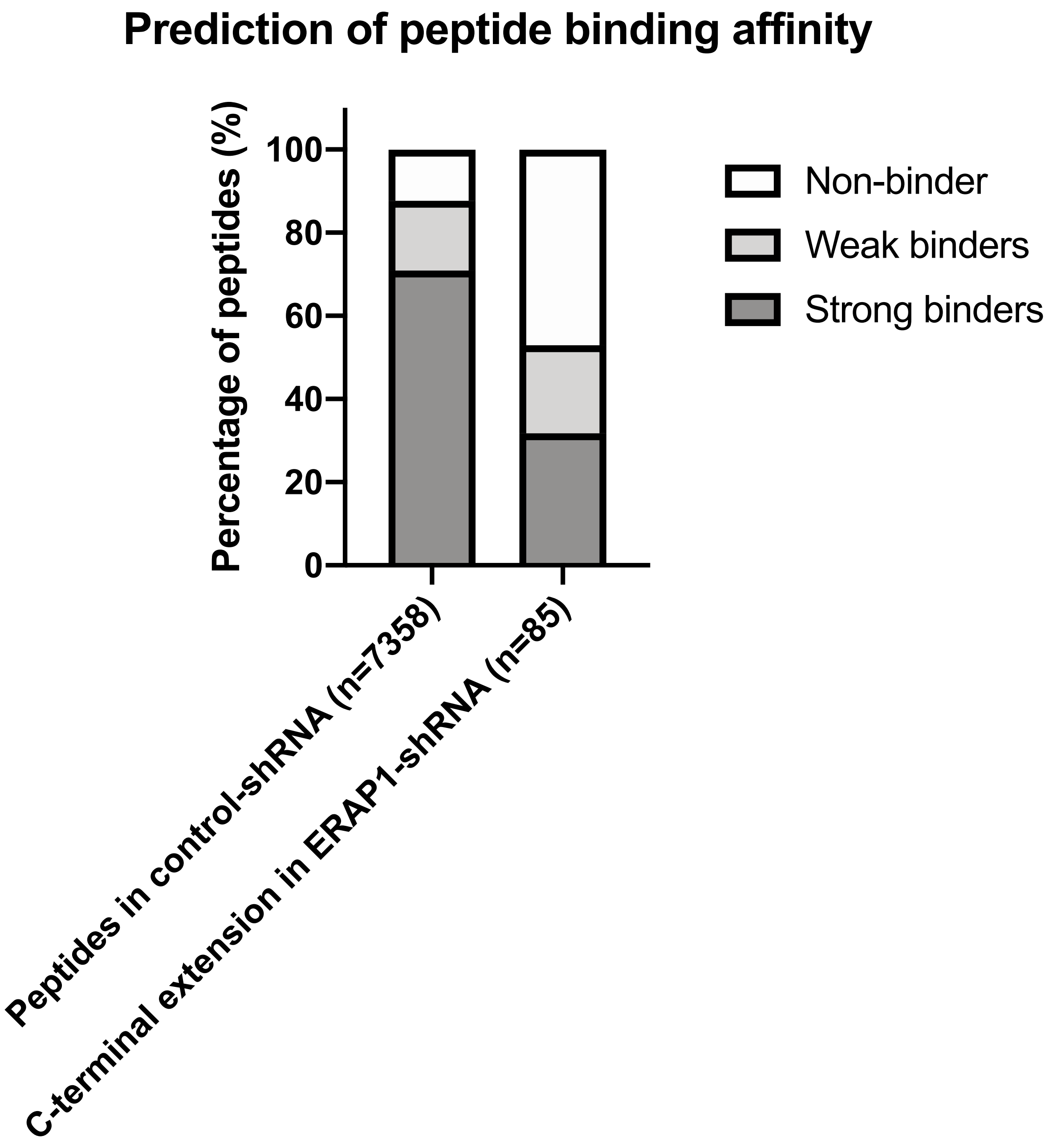

Figure S9. Predicted HLA-B*51:01 binding affiniy of C-terminally extended peptide eluted in ERAP1-sielnced cells. NetMHCpan4.0 was used for in silico prediction. Peptides eluted in Exp-1 was used for analysis.

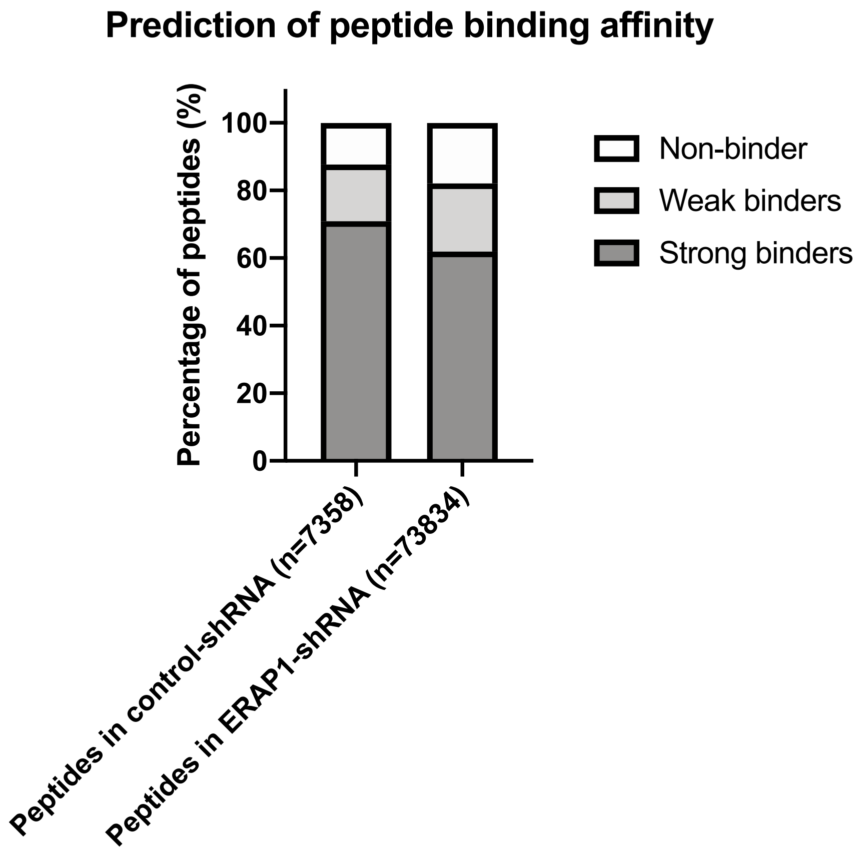

Figure S10. Predcited binding affiniy of peptides eluted in ERAP1-competent and -silenced cells. NetMHCpan4.0 was used for in silico prediction. Peptides eluted in Exp-1 was used for analysis.

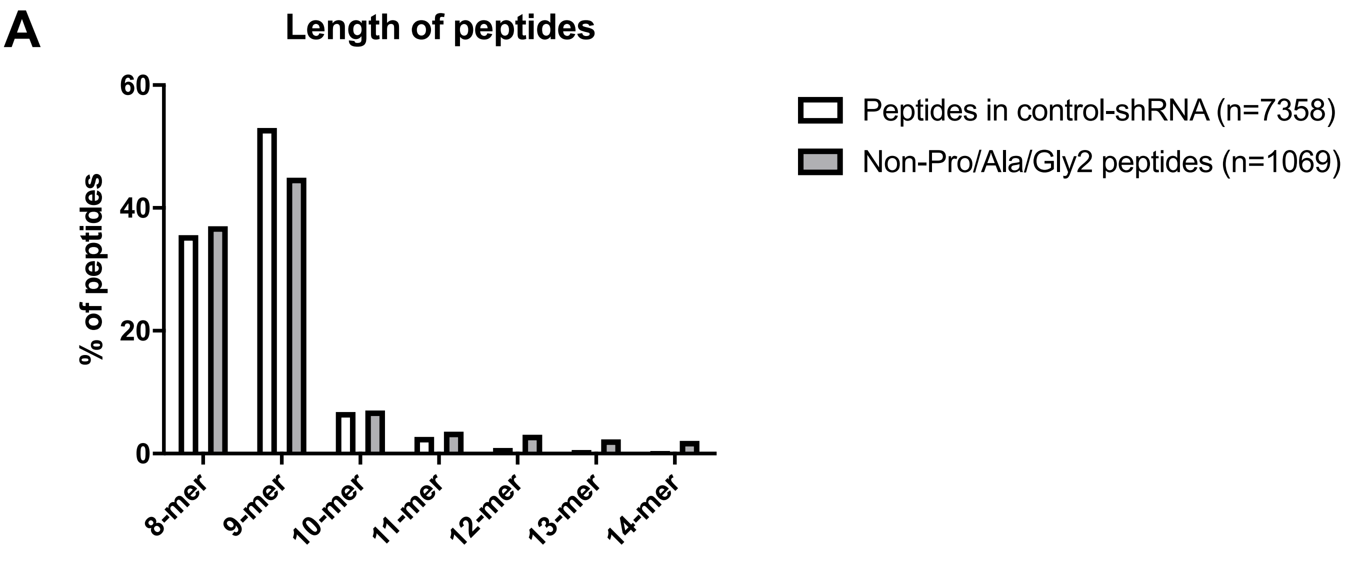

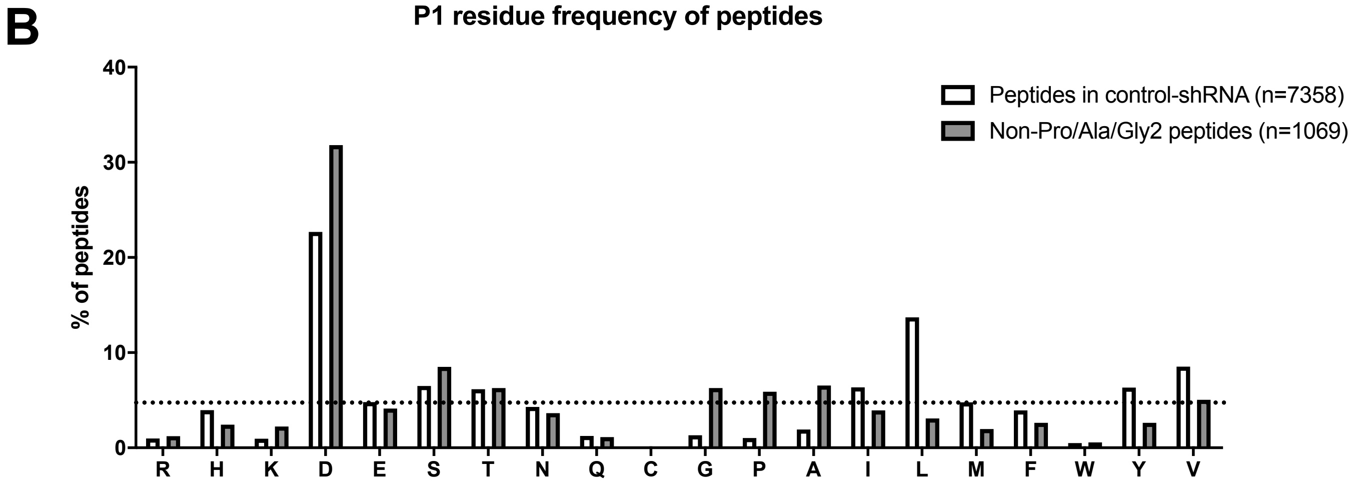

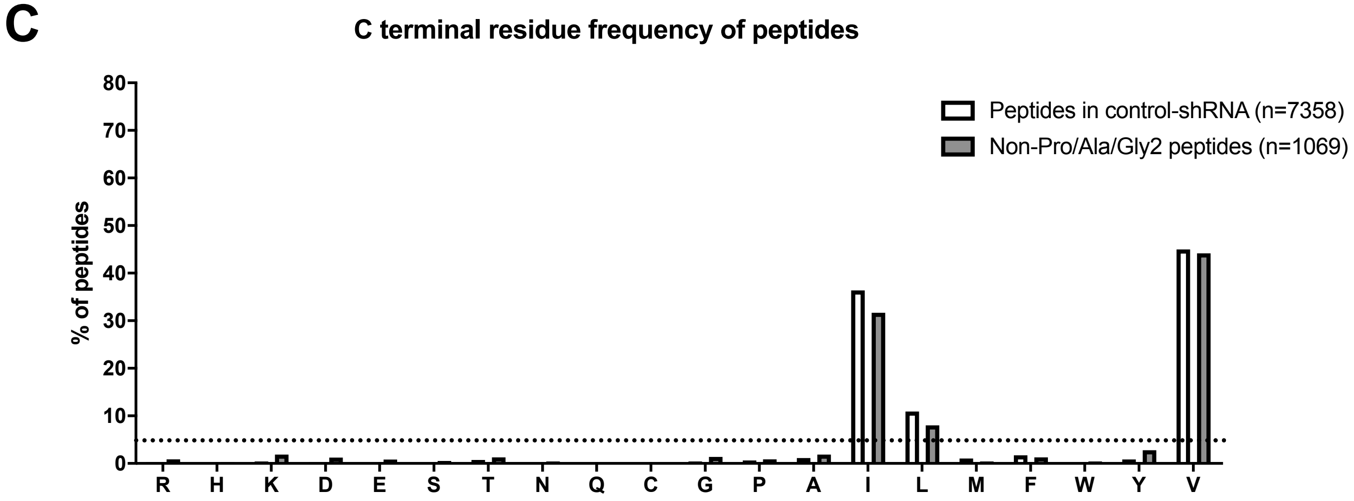

Figure S11. Length and peptide motif of non-Pro/Ala/Gly2 peptides. Peptides eluted in Exp-1 was used for analysis.
